# Isolation and Identification of Wild Yeast from Malaysian Grapevine and Evaluation of Their Potential Antimicrobial Activity against Grapevine Fungal Pathogens

**DOI:** 10.3390/microorganisms9122582

**Published:** 2021-12-13

**Authors:** Simin Sabaghian, Giacomo Braschi, Lucia Vannini, Francesca Patrignani, Nurul Hidayah Samsulrizal, Rosalba Lanciotti

**Affiliations:** 1 Department of Agricultural and Food Sciences, University of Bologna, Piazza Goidanich 60, 47521 Cesena, Italy; simin.sabaghian2@unibo.it (S.S.); lucia.vannini2@unibo.it (L.V.); francesca.patrignani@unibo.it (F.P.); rosalba.lanciotti@unibo.it (R.L.); 2 Interdepartmental Center for Industrial Agri-Food Research, University of Bologna, Piazza Goidanich 60, 47521 Cesena, Italy; 3 Department of Plant Science, Kulliyyah of Science, International Islamic University Malaysia, Kuantan Campus, Kuantan 25200, Malaysia; hidayahsamsulrizal@iium.edu.my

**Keywords:** grapevines, volatile organic compounds, plant pathogens, epiphytic yeast, antifungal activity, biocontrol agents, *Starmerella bacillaris*, *Metschnikowia pulcherrima*

## Abstract

Pathogenic fungi belonging to the genera *Botrytis*, *Phaeomoniella*, *Fusarium*, *Alternaria* and *Aspergillus* are responsible for vines diseases that affect the growth, grapevine yield and organoleptic quality. Among innovative strategies for in-field plant disease control, one of the most promising is represented by biocontrol agents, including wild epiphytic yeast strains of grapevine berries. Twenty wild yeast, isolated and molecularly identified from three different Malaysian regions (Perlis, Perak and Pahang), were evaluated in a preliminary screening test on agar to select isolates with inhibition against *Botrytis cinerea.* On the basis of the results, nine yeasts belonging to genera *Hanseniaspora*, *Starmerella*, *Metschnikowia*, *Candida* were selected and then tested against five grape berry pathogens: *Aspergillus carbonarius*, *Aspergillus ochraceus*, *Fusarium oxysporum*, *Alternaria alternata* and *Phaeomoniella chlamydospora.* *Starmerella bacillaris* FE08.05 and *Metschnikowia pulcherrima* GP8 and *Hanseniaspora uvarum* GM19 showed the highest effect on inhibiting mycelial growth, which ranged between 15.1 and 4.3 mm for the inhibition ring. The quantitative analysis of the volatile organic compound profiles highlighted the presence of isoamyl and phenylethyl alcohols and an overall higher presence of low-chain fatty acids and volatile ethyl esters. The results of this study suggest that antagonist yeasts, potentially effective for the biological control of pathogenic moulds, can be found among the epiphytic microbiota associated with grape berries.

## 1. Introduction

Grapevines (*Vitis vinifera*) are commonly associated with a temperate climate, but over the past decades, a few varieties have been inbred or found to grow well in a tropical climate [1]. In Malaysia, *V. vinifera* grapes for commercial use are produced mainly in Perlis, Perak and Pahang provinces. In 2013, the cultivation area with grapevines was estimated to be 6.6 ha. with 228.5 MT production [2]. In wet tropical areas, however, a successful planting of grapevines will depend on several factors, including the use of greenhouse and pesticides, to protect the vines from rain and fungus outbreaks.

The uncontrolled proliferation of pathogenic fungi belonging to the genera *Botrytis*, *Phaeomoniella*, *Fusarium*, *Alternaria* and *Aspergillus* are responsible for vine diseases that affect plant growth, grapevine yield and organoleptic quality, consequently causing economic losses [3].

The use of synthetic fungicides is effective for the in-field management of grapevine diseases [4], but their intense use has extensive negative effects on ecosystems. Disruptive effects can impact the ecological relationship between the different species able to colonize grapevines and stimulate the selection of resistant pathogen populations to synthetic agents [5].

The increased public concern over the harmful effect of synthetic agents used for crop disease management on the environment, in addition to the restrictions imposed by governmental organizations like the European Union (UE) (Directive 2009/128/EC) [6], have stimulated researchers towards the development of innovative and sustainable systems for harvest crop disease control [7].

Among these innovative and eco-friendly solutions, the use of biopesticides is promising. Biopesticides could be defined as biocontrol agent inhabitants of the same ecological niche as crop pathogens able to counteract their habits and growth [8,9]. Grapevines represent a great source of the microbial community, including yeasts, which are responsible for the safety, quality, and yield of products [10]. Moreover, the grapevine microbiome plays an important role in plant growth, especially in resistance to various types of pathogens [7]. The biodiversity of microorganisms on grapevine berries has been widely studied [11,12,13].

The naturally occurring surface microbiota of grape berry is constituted by a combination of wild yeasts, mainly belonging to non-*Saccharomyces* genera, including *Hanseniaspora*, *Candida*, *Metschnikowia*, *Pichia*, *Zygoascus* and *Issatchenkia* [12,14] that have a significant effect on the health and quality of fruit berries and may have a great impact on the wine-making process as well [10].

Due to their ability to colonize grapevine wound sites, simple nutritional demand, and good rate of growth, epiphytic naturally occurring non-*Saccharomyces* yeasts on grape berries has been largely studied as potential biocontrol agents [8,15,16].

Different non-*Saccharomyces* species, including those belonging to the genera *Aureobasidium*, *Candida*, *Kloeckera*, *Metschnikowia*, *Pichia*, *Saccharomyces*, *Rhodotorula* and *Wickerhamomyces,* have been reported as reducers of fungal pathogens (i.e., *Botrytis cinerea*). Furthermore, they have an impact on fruits through different mechanisms, including nutrient/space competition [17], iron deficiency [18,19], enzymes related to cell wall degradation [19], tolerance to reactive oxygen species [19,20], biofilm production [18] as well as host resistance induction against phytopathogen by phytoalexin production [18] or synthesis of pathogenesis-related proteins [18,21,22]. In this context, the major aim of the presented work was to perform an ecological study on the grape-berry yeast population associated with grape berries from three different regions of Malaysia: Perlis, Perak and Pahang in order to find grape-berry epiphytic yeasts to be used as potential in-field biocontrol agents. For this, isolated indigenous yeasts were characterized in order to assess their in vitro ability to counteract the growth of several grapevines phytopathogens, such as *Botrytis cinerea*, *Phaeomoniella chlamydospora*, *Fusarium oxysporum*, *Alternaria alternata*, *Aspergillus carbonarius* and *Aspergillus ochraceus.* In addition to understanding the mechanisms at the base of the yeast antifungal activities, the strains volatile organic compound profiles were also investigated.

## 2. Materials and Methods

### 2.1. Epiphytic Yeast Strain Isolation and Taxonomic Classification

Epiphytic yeast strains were isolated from grape berries, collected in three different Malaysian regions (Perlis, Perak and Pahang) before harvest in March 2020.

Yeasts were collected by washing grape samples using NaCl saline solution (0.9% p/v). The resulting supernatants were serially diluted (1:10) in the same saline solution and plated on Malt extract Agar (Sigma-Aldrich, Milan, Italy), smented with 200 mg/L of chloramphenicol (Merck, Darmstadt, Germany).

Plates were incubated at 25 °C for 48 h, and the selection of colonies with different morphologies was randomly completed. To obtain pure isolates, single colonies were streaked on MA plates. Purification was repeated at least three times or until all the colonies on the streaked isolate had the same morphology. Isolates were stored at −80 °C in YPD broth (yeast extract 10 g/L, bacteriological peptone 20 g/L and dextrose 20 g/L) added with 25% glycerol. Before each trial, the isolated yeast strains were cultured 2 times in YPD broth and aerobically incubated for 24 h at 25 °C (PH: 5.5).

Extraction of total DNA was conducted by a QIAquick^®^ Genomic Extraction Kit (Qiagen, Hilden, Germany), following the manufacturer’s instructions. Determination of the DNA purity and yields were done by NanoDrop ND1000 UV–Vis Spectrophotometer (Thermo Scientific, Waltham, MA, USA). For all the samples, the yields were approximately 130 ng/μL, and only samples with a ratio of 260_nm_/280_nm_ between 1.9 and 2.1 were used for the polymerase chain reaction.

The total DNA extracted was then used to amplify the internal transcribed spacer region (ITS) that comprises the highly conserved genomic region of ribosomal 5.8S, among two variable zones ITS1 and ITS2. Amplification was carried out by polymerase chain reaction (PCR) using ITS1 (5′- TCCGTAGGTGAACCTGCGG -3′) and ITS4 (5′- TCC TCC GCT TAT TGA TAT GC -3′) with primers as described by [22]. Each 25 μL PCR reaction mixture contained 2.5 μL of 10X reaction buffer, 0.75 mM MgCl_2_ 0.5 mM of each primer, 0.2 mM deoxynucleotides triphosphates (dNTPs), 0.2 U/μL Amplibiotherm Taq DNA Polymerase and 1 μL of total genomic DNA. Primers were purchased from MWG Biotech (, Ebersberg, Germany), while all the PCR reagents were from AURA Biotechnologies Pvt Ltd., Chennai, India. The PCR conditions were as follows: 95 °C for 5 min (initial denaturation) followed by 35 cycles of 95 °C for 1 min (denaturing), 55.5 °C for 2 min (primers annealing), 72 °C for 2 min (elongation). After that, a post-elongation step was performed at 72 °C for 5 min. Amplicons were purified with a QIAquick PCR Purification kit (Qiagen) according to manufacturer specifications and sent to sequencing services at Beijing Genomics Institute (BGI, Shenzhen, China). Obtained sequences were edited with MEGA6 software v2013, and comparisons were made with already published sequences available at GenBank database in NCBI as a reference sequence (National Centre of Biotechnology Information, http://www.ncbi.nlm.nih.gov/ accessed on 1 July 2020) using BLAST (Basic Local Alignment Search Tool). The consistent homologous sequences were aligned by the CLUSTALX 1.8 [23]. Multiple sequence alignments of nt sequences were used for the construction of phylogenetic trees using the neighbour-joining method [24], p-distance method [25] and bootstrap consisting of 1000 pseudo-replicates and finally evaluated using the interior branch test method with MEGA v.6.06 [26] software.

### 2.2. Pathogen Mould Strains and Growth Conditions

Grapevine pathogen moulds used in this experimentation were *Botrytis cinerea*, *Phaeomoniella chlamydospora*, *Fusarium oxysporum*, *Alternaria alternata*, *Aspergillus carbonarius* and *Aspergillus ocharceus*. All the moulds tested were provided by the Department of Science IIUM, Kuantan, Malaysia. Before the experiments, to obtain sporulating colonies, they were cultured for two weeks on Malt extract agar (Oxoid, Thermofisher, Milan, Italy) at 25 °C. From each plate, after incubation, spores were collected using NaCl 0.9% saline solution (5 mL). To remove the mycelial mass, conidia suspensions were filtered on 0.45 μm cutoff diameter filtering membranes, and conidia suspension concentrations were adjusted to give approximately 10^6^ spores/mL. Spore suspensions were stored at 4 °C until used for antifungal assays.

### 2.3. Antifungal In-Vitro Assays

The antifungal activity of grapevine yeast isolates against *Botrytis cinerea*, *Phaeomoniella chlamydospora*, *Fusarium oxysporum*, *Alternaria alternata*, *Aspergillus carbonarius* and *Aspergillus ocharceus* were evaluated in-vitro by the agar-well-diffusion method, as described by [27], with some modifications. Briefly, for each mould, 1 mL of conidial suspension was transferred into an empty petri dish and then covered with 14 mL of sterile malt extract agar cooled at 40 °C. Plates were gently shaken in order to diffuse conidia inoculants, and when the media was solidified, in each plate, an inoculation well (approx. 6 mm ø) was aseptically punched with a tip. Each well was inoculated with 50 µL of the yeast isolate cell-suspension cultured as previously described. Plates were incubated at 25 °C for 7 days. At the end of incubation, yeast antifungal activities were expressed as millimeters of inhibition ring (mm IR).

**Figure 1:**
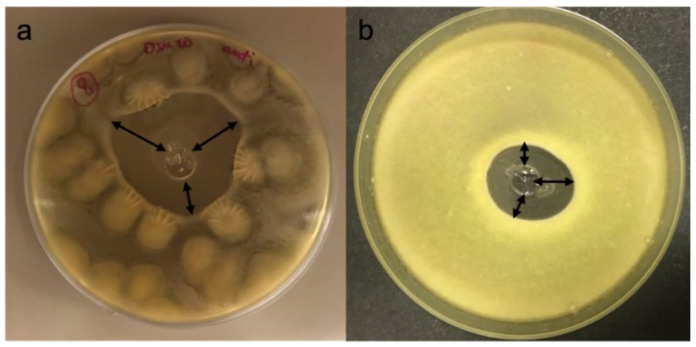
Schematic view of the measurement of inhibition of fungal mycelial growth. Three different inhibition rings (mm IR) were measured for each plate considered, as illustrated with black double arrows. (**a**) *Botrytis cinerea*; (**b**) *Alternaria alternata*.

The inhibition ring was measured, using a calliper, from the edge of the inoculation well to the innermost mould growth perimeter, as shown in Figure 1. Antifungal activities were tested in triplicates using plates inoculated with NaCl saline solution (0.9% p/v) as a negative control.

### 2.4. Yeast Volatile Organic Compound (VOC) Profiles

The yeast volatile organic compound (VOC) compositions were qualitatively and quantitatively evaluated with head space solid-phase microextraction using a gas chromatograph coupled with a mass spectrometer detector (GCMS-SPME). Analyses were performed after 6 days of growth at 25 °C in liquid media (malt extract broth) of *M. pulcherrima* GP8, *S. bacillaris* FE08.05, *H. opuntiae* GA22, *H. pseudoguilliermondii* GP14, *H. lanchancei* GM32, *H. guilliermondii* GA1, *H. uvarum* GM19, *H. opuntiae* GM10 and *C. awuaii GM3*. A CAR/PDMS 75μm fibre (SUPELCO, Bellafonte, PA, USA) was used to perform the solid-phase microextraction (SPME). The samples (5 mL) were placed in vials and incubated for 10 min at 45 °C. Then, the fiber was exposed to the headspace of the vial for 30 min at 45 °C. The volatile molecules adsorbed were desorbed in the gas chromatograph (GC) injector port in splitless mode at 250 °C for 10 min. The headspace of the volatile compounds was analyzed using chromatography (GC) 6890 N, Network GC System with mass spectrometry (MS) 5970 MSD (Aglient technologies, Milan, Italy). The column used was J&W CP-Wax 52 (50 m × 320 μm × 1.2 μm) (Aglient technologies, Milan, Italy). The initial temperature was 40 °C for 1 min and then increased by 4.5 °C/min up to 65 °C. After that, the temperature increased by 10 °C/min up to 230 °C and remained at this temperature for 17 min. The gas-carrier was helium at 1.0 mL/min flow. Compounds were identified by comparison based on the NIST 11 (National Institute of Standards and Technology) database, while the quantitative analysis was performed with the internal standard method using 4-methyl-2-pentanol (6 mg/L) and expressed as equivalent ppm (ppm eq.). For each compound detected, the ppm eq. represents the amount of compound present in the headspace in dynamic equilibrium with the aqueous phase. The chemical analyses were performed in triplicate and are expressed as means.

### 2.5. Detached Berry Antifungal Assay

Yeast isolates were tested for antagonistic activity against *A. carbonarius* in a detached berry test as described previously by [28], with some modifications. Briefly, the selected yeast strains were grown in liquid culture of Malt extract broth (Oxoid, Thermofisher, Milan, Italy) without stirring for 48 h at 25 °C. Mature grape berries of the Red Globe variety detached from bunches were sanitized on the surface using 1% commercial sodium hypochlorite for 15 min and rinsed with sterile deionized water, and dipped inside yeast 48 h cultures. After 4 h of incubation at 25 °C, berries were air-dried, and a wound (about 2 mm diameter) was made on each berry with a sterile needle. The wound was spot-inoculated with 20 µL of *A. carbonarious* conidial suspension (approx. 10^6^ conidia/mL). Berries were incubated at 25 °C for 10 days. The inhibition expressed as *A. carbonarius* diameter of growth (ø. mm) was monitored daily using a calliper.

### 2.6. Statistical Analysis

Data were processed using the SPSS software tool (Version 26). Yeast antifungal in vitro properties against the selected phytopathogens moulds, as well as mycelial growth inhibition on detached berries, were considered statistically different (*p* < 0.05) based on ANOVA and TUKEY HSD post-hoc tests.

To obtain a visual overview of the volatile organic compounds of the selected yeast isolates, principal component analysis (PCA) was used. Quantitative data of VOC profiles were analyzed using ANOVA followed by DUNCAN’s tests (*p* < 0.05).

## 3. Results

### 3.1. Molecular Analysis of Yeast Isolates

**Figure 2:**
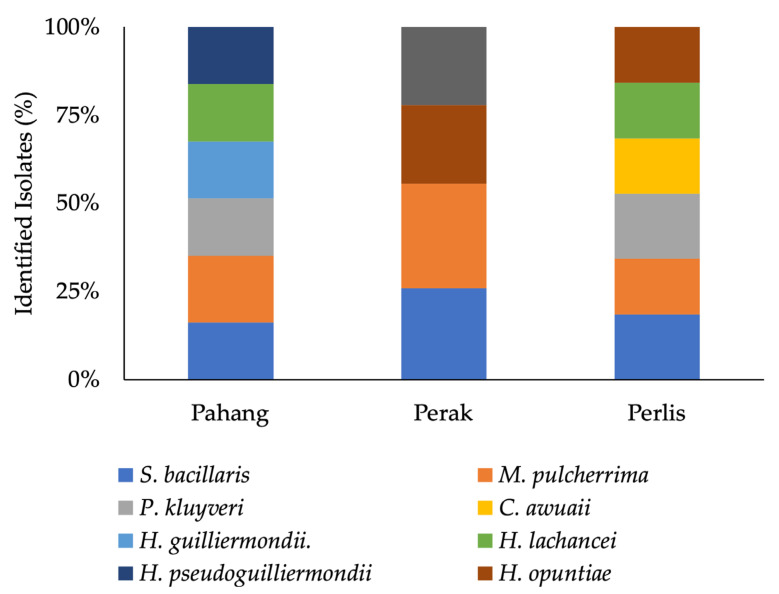
Occurrence of isolated yeast in three main regions of sampling (Pahang, Perak and Perlis). Vertical axis shows the occurrence percentage.

A total of 20 yeasts were isolated from grape samples obtained from the three Malaysian sampling regions Perlis, Perak and Pahang. Isolated yeasts were identified according to the nucleotide sequences of the ITS region. As shown in Figure 2, the identification based on the ITS region sequences revealed a dominant non-*Saccharomyces* indigenous population. Specifically, isolated yeast belonged to eight different genera, including *Hanseniaspora*, *Starmerella*, *Metschnikowia*, *Pichia*, *Candida*. All the sampling regions were characterized by the presence of *Metschnikowia pulcherrima* and *Starmerella bacillaris* and a strain belonging to the *Hanseniaspora* genus.

**Figure 3:**
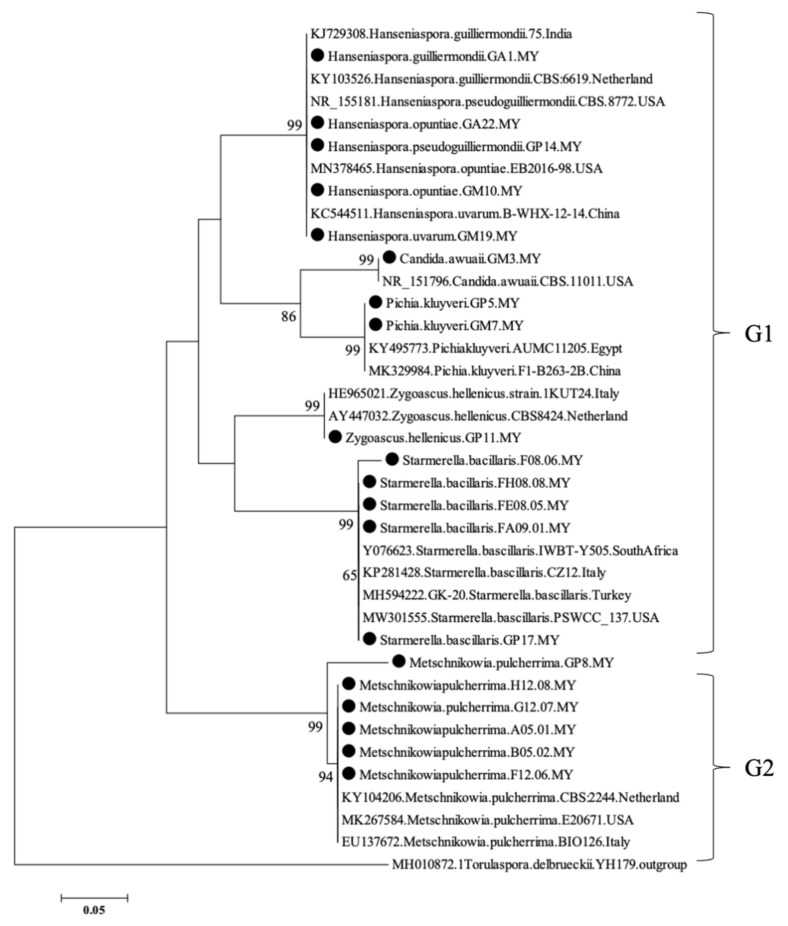
Phylogenetic tree constructed on the sequence alignment of ITS1 and ITS4 regions representing isolated yeasts of grapevine berry and their homologue-related species. Black circles represented the isolated yeast in this study. The phylogenetic tree is inferred from the “Neighbour joining method” and numbers on branches are derived from bootstrap resembled datasets, indicated as percentage of support from 1000 bootstrap replications. Branch lengths represent bootstrap values. Nodes with less than 70% bootstrap support were collapsed. *Torulaspora delbrueckii* was used as an outgroup species to root the tree. The bar represents 0.05 changes per site.

Within the *Hanseniaspora* genus, the species isolated were *H. lachancei*, *H. opuntiae*, *H. guilliermondii*, *H. pseudoguilliermondii* and *H. uvarum*. Yeast isolated from the Pahang and Perlis regions showed a higher variability compared to the ones isolated from Perak. In addition to the species already mentioned, grape berries from the Pahang and Perlis regions were characterized by the presence of *Pichia kluyveri* and *Candida awuaii* strains. Twenty isolates obtained in this study grouped with representative type strains of known yeast species in the phylogenetic tree with high nucleotide similarity (Table 1), including previously described yeast species (Figure 3). The phylogenetic tree based on the complete nucleotide sequence of the ITS region generated two different groups, while the identified isolates scattered in both groups 1 and 2, close to isolates of different distances, indicating the variation and long-distance migration in Malaysian isolates and other countries (Figure 3).

### 3.2. In-Vitro Antifungal Assays

**Figure 4:**
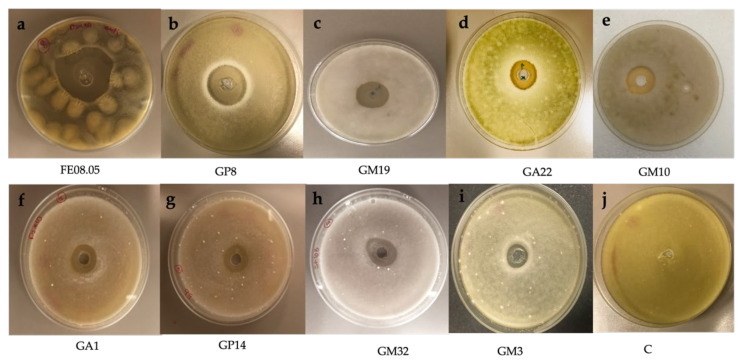
Mycelial growth inhibition of 9 different isolated yeast on *Botrytis cinerea*. (**a**) *Starmerella bacillaris* FE08.05, (**b**) *Metschnikowia pulcherrima* GP8, (**c**) *Hanseniaspora uvarum* GM19, (**d**) *Hanseniaspora opuntiae* GA22, (**e**) *Hanseniaspora opuntiae* GM10, (**f**) *Hanseniaspora guilliermondii* GA1, (**g**) *Hanseniaspora pseudoguilliermondii* GP14, (**h**) *Hanseniaspora lanchancei* GM32, (**i**) *Candida awuaii* GM3. (**j**) Negative control (C) filled with NaCl saline solution (0.9% p/v).

**Figure 5:**
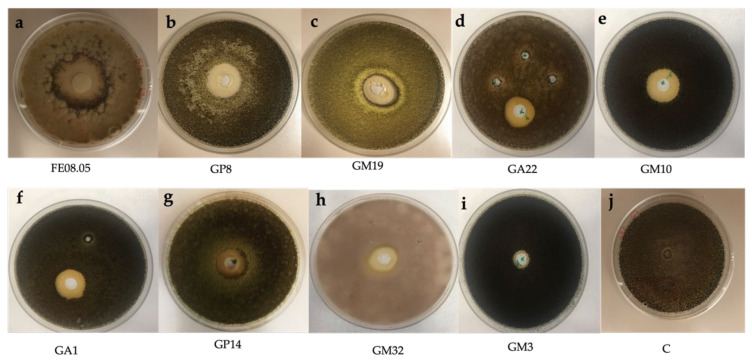
Mycelial growth inhibition of 9 different isolated yeast on *Aspergillus carbonarius*. (**a**) *Starmerella bacillaris* FE08.05, (**b**) *Metschnikowia pulcherrima* GP8, (**c**) *Hanseniaspora uvarum* GM19, (**d**) *Hanseniaspora opuntiae* GA22, (**e**) *Hanseniaspora opuntiae* GM10, (**f**) *Hanseniaspora guilliermondii* GA1, (**g**) *Hanseniaspora pseudoguilliermondii* GP14, (**h**) *Hanseniaspora lanchancei* GM32, (**i**) *Candida awuaii* GM3. (**j**) Negative control (C) filled with NaCl saline solution (0.9% p/v).

Twenty yeast isolates were tested for their potential in vitro antifungal activity. First, a preliminary screening was performed against the phytopathogen *B. cinerea* (Figure 4) and only strains characterized by an inhibitory activity were also tested against other selected pathogens (*A. carbonarius*, *A. ochraceus*, *F. oxysporum*, *A. alternata* and *P. chlamydospora)* (Figure 5). Among 20 yeast strains tested, all *Pichia kluyveri* strains had no antagonistic activity against the selected moulds (data not shown), and only nine isolates had the ability to reduce *B. cinerea* mycelial growth (Table 2).

Yeast strains with anti-mycelial activities were *S. bacillaris* FE08.05, *M. pulcherrima* GP8, *H. uvarum* GM19, *H. opuntiae* GA22, *H. opuntiae* GM10, *H. guilliermondii* GA1, *H.* *lachancei* GM32, *H. pseudoguilliermondii* GP14 and *C. awuaii* GM3 (Table 3). *S. bacillaris* FE08.05 and *M.* *pulcherrima* GP8 showed the highest inhibitory effects against all the pathogens tested, except in the case of *P. chlamydospora*, which was not inhibited from any yeast tested (Table 3). The inhibitory effects of these strains were similar, and the yeast significantly (*p* < 0.05) affected the growth of *B. cinerea* (Figure 4) with inhibition rings of 15.1 mm, 12.4 mm and 10.8 mm, respectively.

*S. bacillaris* FE08.05 and *M. pulcherrima* GP8 also strongly inhibited the growth of *A. carbonarius* (14.2 and 10.2 mm IR) (Figure 5) and *A. ocharaceus* (12.2 and 8.2 mm IR), while only *S. bacillaris* FE08.05 strongly reduced the growth of *A. alternata* (15.8 mm IR) and *F. oxysporum* (10.5 mm IR) (Table 3).

Among the *Hanseniaspora* species assessed, *H. uvarum* isolate GM19 showed a similar inhibition pattern to *S. bacillaris* FE08.05 and was more active against *B. cinerea* and *A. carbonarius* (Figure 4 and Figure 5). The inhibitory ring against the moulds ranged between 6.2 and 10.8 mm. On the other hand, *H. opuntiae* GM10, *H. opuntiae* GA22, H. *guilliermondii GA1*, *H. lanchancei* GM32 and *H. pseudoguilliermondii* GP14 were less effective in inhibiting the mycelial growth of the selected pathogens. *C. awuaii* GM3 did not show a strong inhibitory effect against the selected phytopathogens (Table 3).

### 3.3. Volatile Organic Compound Profiles

**Figure 6:**
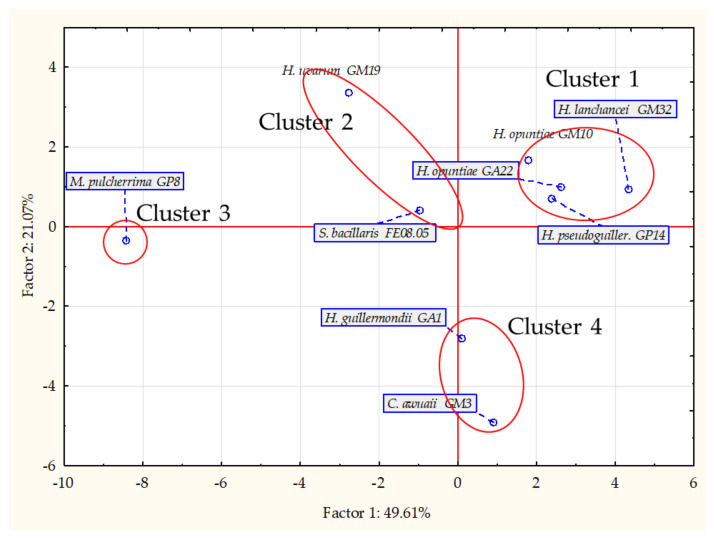
Principal component analysis loading plot of volatile organic compounds (VOCs) of the selected yeast strains with antifungal properties after 6-days of growth in malt extract broth at 25 °C.

In Figure 6 we show the principal component analysis (PCA) loading plots of yeast volatile organic profiles (VOCs) produced after 6 days of growth on malt extract broth. The principal component analysis allowed the discrimination of yeast in relation to their VOCs produced during the growth in relation to their species and genus (Figure 6).

Samples were mapped in the space spanned by the first two principal components, PC1 and PC2. The analysis allowed us to explain over 70% of the total variability observed (Figure 6). PC1 accounted for 49.61% of the total variability, and PC2 for 21.07%. Except for *H. guilliermondii* GA1 and *H. uvarum* GM19, *Hanseniaspora* genus strains grouped alongside the variable plane defined by PC1 and PC2 (Cluster 1). *M. pulcherrima* GP8 was clearly separated, along the PC1, from the other species (Cluster 3), while *C. awuaii* GM3 and *H. guilliermondii* GA1 formed a cluster separated from the other species along the PC2. *S. bacillaris* FE08.05 separated along the PC1 from *M. pulcherrima* GP8 clustered next to *H. uvarum* GM19 (Cluster 2).

VOC profiles were mainly characterized by alcohols, organic acids and esters (Table 4). After 6 days of growth, *M. pulcherrima* GP8 and *S. bacillaris* FE08.05 produced the highest level of isoamyl (8.69 and 8.99 ppm) and phenylethyl alcohol (10.91 and 3.16 ppm) (Table 4). These strains also produced moderate amounts of other VOCs, including low-molecular-weight organic acids and esters ranging from 0.13 to 1.80 ppm (Table 4). Among *Hanseniaspora* strains, the production of VOCs belonging to *H. uvarum* GM19 was notable. This strain produced comparable amounts of isoamyl to *S. bacillaris* FE08.05 (8.07 ppm) and phenylethyl (2.51 ppm) alcohols. *C. awuaii* GM3 and *H. guilliermondii* GA1 showed an overall reduced production of VOCs (Table 4).

### 3.4. Detached Berry Antifungal Assay

**Figure 7:**
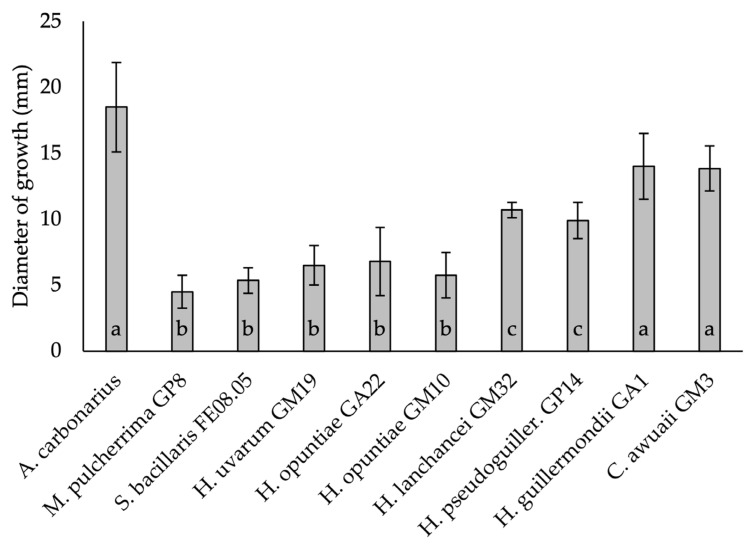
Evaluation of the antifungal activity (growth ø. mm) after 10 days of incubation at 25 °C of the selected yeast strains against *Aspergillus carbonarius* on the detached berry. Results are the mean of 3 replicates. Different letters indicate significantly (*p* < 0.05) different growth of *Aspergillus carbonarius* on the base of ANOVA and TUKEY HSD tests.

The nine yeast isolates showing in vitro antifungal activity were evaluated for their efficacy to inhibit the growth of *A. carbonarius* on detached berries (Figure 7). Among the tested strains, after 10 days of incubation, *M. pulcherrima* GP8, *S*. *bacillaris* FE08.05, *H. uvarum* GM19, *H. opuntiae* GA22 and *H. opuntiae* GM10 had a similar and significant (*p* < 0.05) inhibition against *A. carbonarius*.

In the control berry batch, *A. carbonarious* reached 18.5 mm of growth diameter (ø. mm), while in the presence of the yeast strains, the mycelial growth ranged between 4.5 and 6.8 mm. Moderate inhibitory effects, compared to the other stains, were also observed for *H. lachancei* GM32 (10.7 mm) and *H. pseudoguilliermondii* GP14 (9.9 mm), while *H. guilliermondii* GA1 and *C. awuaii* GM3 had no effects on the mycelial growth inhibition (Figure 7).

## 4. Discussion

The main aim of the presented work was to isolate epiphytic yeast from *V. vinifera* grape berries grown in different regions of Malaysia (Perlis, Perak and Pahang) and evaluate their ability to inhibit the mycelial growth of six selected grapevine phytopathogens: *Botrytis* cinerea, *A. carbonarius*, *A. ochraceus*, *F. oxysporum*, *A. alternata* and *P. chlamydospora*. The major component of the microbiota on the surface of plants, fruits and vegetables is represented by epiphytic yeasts [29]. Yeasts are evolutionarily adapted to such ecosystems and are able to colonize in many different environmental conditions, plants and grape surfaces or wounds [29].

Many ecological studies have revealed that epiphytic yeasts present on grape berries belong to non-*Saccharomyces* genera, including *Hanseniaspora*, *Candida*, *Metschnikowia*, *Pichia*, *Zygoascus* and *Issatchenkia* [11,12,13,14]. According to the literature, yeast species isolated from the three different Malaysian regions belongs to the genera: *Hanseniaspora*, *Starmerella*, *Metschnikowia*, *Pichia*, *Candida.*

However, epiphytic yeast populations isolated from the Pahang and Perlis regions were characterized with a high variability compared to the Perak region.

Although yeasts were isolated simultaneously, the different grapevine plants physiological status could influence the indigenous epiphytic yeast population [11].

The in-field management of plant pathogens using naturally occurring epiphytic yeasts represent one promising and sustainable strategy to reduce chemicals and pesticides commonly used to achieve these purposes. The results presented in this study suggest that antagonist yeasts with the potential to control *B. cinerea*, *A. carbonarius*, *A. ochraceus*, *F. oxysporum*, *A. alternata* and *P. chlamydospora* on grapes can be found among the microflora associated with the berries. Generally, the selected yeasts have antagonistic activity against the selected pathogen fungi and the ability to inhibit mycelial growth was more frequently observed. The highest effect on inhibiting mycelial growth was shown by *S. bacillaris* FE08.05, which was able to strongly reduce mycelial growth in all tested fungi, while the next significant inhibition belongs to the *M. pulcherrima* GP8 and *H. uvarum* GM19 strains (Table 3).

In addition, these stains were characterized by the highest anti-mycelial growth activity against *A. carbonarius* when inoculated in detached grapevines berries (Figure 6).

*S. bacillaris* is available in oenological environments regarding its osmotolerant nature and is periodically detected on fruit surfaces, *Drosophila spp.* and soil [30]. Several surveys have largely demonstrated that its use, together with selected *Saccharomyces cerevisiae,* in mixed culture fermentations enhanced the analytical composition and aroma profile of wine [30,31]. However, few investigations have analyzed the antifungal activity of *S. bacillaris* strains on post-harvest fruits. Some researchers have shown the inhibitory activity of *S. bacillaris* strains against *B. cinerea* on grapes in vineyards, in line with our results [32,33].

*S. bacillaris* has been introduced as a safe microorganism with the potential ability to be used as a biocontrol agent against different food pathogens [33]. Junior et al. [33] reported that there is no pathogenicity factor for human health regarding *S. bacillaris* as a biocontrol agent. *S. bacillaris* FE08.05 also successfully controlled the growth of *A. alternata* (Table 3). The biocontrol of *A. alternata* could be the result of these yeast species colonizing wound sites, which implies competitive mechanisms [34]. A similar inhibition was also observed using *M. pulcherrima* GP8 and *H. uvarum* GM19.

Guinebretière et al. [35] reported *M. pulcherrima* showing an inhibitory effect against *Botrytis cinerea* in grape and strawberry.

Mycelial growth of *A. alternata* was significantly inhibited by all tested yeasts; again, *S. bacillaris* strain FE08.05, *M. pulcherrima* strain GP8 and *H. uvarum* GM19 were the most effective among others. Stocco et al. [36] indicated that *M. pulcherrima* could be used as a biocontrol agent against *A. alternata* in table grape, which is in line with our results. Moreover, *Aspergillus ochraceus* mycelial growth was significantly inhibited by *S. bacillaris* FE08.05, *M. pulcherrima* GP8.

Furthermore, previous research confirms that yeast *Hanseniaspora opuntiae* HoFs can protect plants against *Botrytis cinerea* and *Corynespora cassiicola* [37]. *Hanseniaspora uvarum* had an intermediate effect on *Phaeomoniella chlamydospora* mycelial growth, and this result is in accordance with Zhang et al. [38], who reported considerable inhibition of the spore germination of *Penicillium digitatum by H. uvarum* Y3 in orange.

This study confirmed that *M. pulcherrima* is able to reduce the growth of *A. carbonarius*, *Aspergillus ochraceus* and *Fusarium oxysporum* growth, which is in line with previous research by Bleve et al. [29] and Turkel et al. [39], who indicated that *M. pulcherrima* is able to reduce *A. niger*, *A. carbonarius* and *Fusarium* spp. growth on agar plates.

The PCA of volatile organic compounds produced during the yeast growth allowed the grouping of the selected strains into four different clusters. In agreement with the presented data, clusters 2 and 3 formed by *M. pulcherrima* GP8, *S. bacillaris* FE 08.05 and *H. uvarum* GM19 were capable of inhibiting the selected grapevine pathogenic moulds.

The quantitative analysis of their VOC profiles highlighted the presence compared to the other strains of higher levels of isoamyl and phenyletyl alcohols and an overall higher presence of other secondary metabolites, including low molecular weight organic acids (acetic, isovaleric, n-caprylic, pelargonic and n-capric acid) and volatile ethyl esters.

Phenyletyl ethanol and isoamyl alcohols successfully demonstrated inhibition of mycelial growth of *Aspergillus flavus* [40,41] and *Aspergillus brasiliensis* [41,42]. Although present in small amounts, short and medium-chain fatty acids and ethyl fatty acids esters can also synergize with higher alcohol antimicrobial activity [43].

Even though further investigations are needed to assess whether these yeast isolates have practical value in the control of other fungi occurring on grapes, the data reported here indicate that these yeasts originated from grapevine itself and can be described as “ecological fungicides” without any effect on the balance of the environment. This could be a motivation for industry and manufacturing sectors to produce biosafety products using those species in the near future. Our outcomes showed that the protentional biocontrol activity is related to the characterization of strain, as observed by Suzzi et al. [17] in a previous study on the antagonistic aptitudes of wine yeasts against plant pathogenic fungi.

## 5. Conclusions

In conclusion, the preliminary results presented in this work highlight the occurrence of epiphytic indigenous yeast on grapes isolated from three different Malaysian regions that can potentially counteract the mycelial growth of several grape berry pathogen moulds. Among the isolated strains, *M. pulcherrima* GP8, *S. bacillaris* FE 08.05 and *H. uvarum* GM19 seems to be the most promising, as highlighted by in vitro antifungals and in detached berry trials. Volatile organic compounds revealed the production from these strains of different volatile antimicrobial compounds, including higher alcohols, low-chain fatty acids and esters. However, more trials are needed.

Since non-*saccharomyces* species, as well as those belonging to the *Metschnikowia* and *Hanseniaspora* genera, could have misidentifications based on ITS sequences, and for these reasons, other genetic identifications based on D1/D2 ribosomal subunits and 26S rRNA sequences will be performed.

Since biological control agent efficacy can vary according to the pathogen’s inoculum level and environmental conditions [44], in-field trials are necessary.

In addition, a deeper knowledge about yeast inhibitory mechanisms is essential for the development of tailor-made strategies that can be more effective and guarantee better performance in the field. For these reasons, non-volatile organic compounds produced by yeast strains during growth will be considered.

Finally, the presented research pinpointed the importance of studying and exploiting natural and indigenous microflora to find sustainable and wild microbial strains, alternatives to engineered ones, able to counteract the main crop pathogens.

## Figures and Tables

**Table 1:** Identification of yeasts with potential for biological control. Values from pairwise sequence comparisons based on the highest sequence identity found in BLAST analysis. E-value (expectation value) represents the number of expected hits of similar quality (score) that could be found by chance.

Isolate	Species Designation	Accession Number/Country/Region	Identity (%)	E-Value
F08.06	*Starmerella bacillaris*	OK329946/Malaysia/Perak	99.28	0.00
FH08.08	*Starmerella bacillaris*	OK329947/Malaysia/Pahang	99.28	0.00
FA09.01	*Starmerella bacillaris*	OK329948/Malaysia/Perak	98.8	0.00
FE08.05	*Starmerella bacillaris*	OK329949/Malaysia/Pahang	99.04	0.00
GP17	*Starmerella bascillaris*	OK329950/Malaysia/Perlis	99.75	0.00
PSWCC_137	*Starmerella bascillaris*	MW301555/USA	Ref-isolate	-
H12.08	*Metschnikowia pulcherrima*	OK329951/Malaysia/Perak	97.93	2 × 10^−162^
A05.01	*Metschnikowia pulcherrima*	OK329952/Malaysia/Perak	97.93	2 × 10^−162^
B05.02	*Metschnikowia pulcherrima*	OK329953/Malaysia/Perak	97.12	2 × 10^−162^
F12.06	*Metschnikowia pulcherrima*	OK329954/Malaysia/Pahang	97.12	2 × 10^−162^
G12.07	*Metschnikowia pulcherrima*	OK329955/Malaysia/Perlis	97.93	2 × 10^−162^
GP8	*Metschnikowia pulcherrima*	OK560819/Malaysia/Perlis	96.43	0.00
E20671	*Metschnikowia pulcherrima*	MK267584/USA	Ref-isolate	-
GM3	*Candida awuaii*	OK329958/Malaysia/Pahang	87.27	1 × 10^−94^
CBS.11011	*Candida awuaii*	NR_151796/USA	Ref-isolate	-
GA1	*Hanseniaspora guilliermondii.*	OK329959/Malaysia/Perlis	99.57	0.00
CBS:6619	*Hanseniaspora guilliermondii.*	KY103526/Netherland	Ref-isolate	-
GM10	*Hanseniaspora opuntiae*	OK329962/Malaysia/Perak	100	0.00
GA22	*Hanseniaspora opuntiae*	OK329963/Malaysia/Pahang	99.85	0.00
EB2016-98	*Hanseniaspora opuntiae*	MN378465/USA	Ref-isolate	-
GP14	*Hanseniaspora pseudoguilliermondii*	OK329961/Malaysia/Perlis	99.41	0.00
CBS.8772	*Hanseniaspora pseudoguilliermondii*	NR_155181/USA	Ref-isolate	-
GM19	*Hanseniaspora uvarum*	OK329964/Malaysia/Pahang	100	0.00
B-WHX	*Hanseniaspora uvarum*	KC544511/China	Ref-isolate	-
GP5	*Pichia kluyveri*	OK329956/Malaysia/Perlis	98.46	0.00
GM7	*Pichia kluyveri*	OK329957/Malaysia/Pahang	99.22	0.00
F1-B263-2B	*Pichi kluyveri*	MK329984/China	Ref-isolate	-
GP11	*Zygoascus hellenicus*	OK329960/Malaysia/Perlis	99.81	0.00
1KUT24	*Zygoascus hellenicus*	HE965021/Italy	Ref-isolate	-

**Table 2:** Yeast strains isolated from grape berries and characterized by in vitro antifungal activity against *Botrytis cinerea*.

Isolate	Designated Species	*B. cinerea* Antifungal Activity
FE08.05	*Starmerella bacillaris*	+
GP8	*Metschnikowia pulcherrima*	+
GM19	*Hanseniaspora uvarum*	+
GA22	*Hanseniaspora opuntiae*	+
GM10	*Hanseniaspora opuntiae*	+
GA1	*Hanseniaspora guilliermondii*	+
GP14	*Hanseniaspora pseudoguilliermondii*	+
GM32	*Hanseniaspora* *lanchancei*	+
GM3	*Candida awuaii*	+

**Table 3:** In vitro inhibition of mycelial growth (diameter of inhibition mm) of *Botrytis cinerea*, *Aspergillus carbonarious*, *Aspergillus ochraceus*, *Alternaria alternata*, *Fusarium oxysporum*, *Phaeomoniella chlamydospora* by *Starmerella bacillaris* (FE08.05), *Metschnikowia pulcherrima* GP8, *Hanseniaspora*, *uvarum* GM19, *Hanseniaspora*, *opuntiae* GA22, *Hanseniaspora opuntiae* GM10, *Hanseniaspora guilliermondii* GA1, *Hanseniaspora* *lachancei* GM32, *Hansenaspora pseudoguilliermondii* GP14 (*H. peseudoguller* GP14) and *Candida awuaii* GM3. Results are the mean of three replicates. For each pathogen considered, different letters indicate significantly (*p* < 0.05) different inhibition on the basis of ANOVA and TUKEY HSD tests.

	Inhibition Ring (mm)								
	*S. bacillaris* FE08.05	*M. pulcherrima*GP8	*H. uvarum* GM19	*H. opuntiae* GA22	*H. opuntiae* GM10	*H. guilliermondii* GA1	*H. lachancei* GM32	*H. pseudoguillier.* GP14	*C. awuaii* GM3
*B. cinerea*	15.1 ^a^ ± 0.4	12.4 ^b^ ± 0.5	10.8 ^c^ ± 0.3	8.1 ^d^ ± 0.3	5.8 ^e^ ± 0.3	3.0 ^g^ ± 0.2	2.0 ^h^ ± 0.2	4.4 ^g^ ± 0.5	6.3 ^e^ ± 0.3
*A. carbonarius*	14.2 ^a^ ± 0.3	10.2 ^b^ ± 0.3	8.2 ^c^ ± 0.3	6.2 ^d^ ± 0.3	3.2 ^e^ ± 0.3	2.2 ^f^ ± 0.3	<1 *	2.1 ^f^ ± 0.1	1.2 ^g^ ± 0.3
*A. ochraceus*	12.2 ^a^ ± 0.3	8.2 ^b^ ± 0.3	3.2 ^e^ ± 0.3	5.9 ^c^ ± 0.1	3.2 ^d^ ± 0.3	2.2 ^f^ ± 0.3	<1	2.1 ^f^ ± 0.1	1.2 ^g^ ± 0.3
*A. alternata*	15.8 ^a^ ± 0.2	6.3 ^b^ ± 0.5	8.2 ^c^ ± 0.3	5.2 ^d^ ± 0.3	2.1 ^e^ ± 0.1	1.1 ^f^ ± 0.1	1.2 ^f^±0.3	5.2 ^d^ ± 0.3	2.2 ^e^ ± 0.3
*F. oxysporum*	10.5 ^a^ ± 0.5	5.4 ^a^ ± 0.4	4.3 ^c^ ± 0.2	1.2 ^d^ ± 0.3	<1	<1	<1	<1	<1
*P. chlamydospora*	<1	<1	<1	<1	1.2 ^a^ ± 0.3	<1	<1	<1	<1

* Under the detection limit (inhibition ring < 1 mm). The letters (a–g) shows mean comparison analysis and same letters means no statistically significant difference between the yeast inhibition zone.

**Table 4:** Volatile organic compounds (VOCs) produced by 6-day-old cultures of the selected strains with antifungal properties. Data are the mean of three replicates. Standard deviation observed ranged between 5 and 7%. Results are the mean of three replicates. For each compound considered, different letters indicate significantly (*p* < 0.05) different amounts based on DUNCAN’s tests.

Yeast Isolate	*ppm eq.*										
	Alcohols				Organic Acids					Esters	
	Isobutyl Alcohol	Isoamyl Alcohol	Phenylethyl Alcohol	Isoprenyl Alcohol	Acetic Acid	Isovaleric Acid	n-Caprylic Acid	Pelargonic Acid	n-Capric Acid	Ethyl Propionate	Lauric Acid, Ethyl Ester
*M. pulcherrima* GP8	0.66 ^a^	8.69 ^a^	10.91 ^a^	1.04 ^a^	0.49 ^a^	1.30 ^a^	0.89 ^a^	0.34 ^a^	0.77 ^a^	-	1.80 ^a^
*S. bacillaris* FE08.05	- *	8.99 ^a^	3.16 ^b^	0.95 ^a^	0.22 ^b^	0.17 ^b^	0.76 ^a^	0.19 ^b^	0.88 ^a^	0.18	0.26 ^b^
*H. opuntiae* GA22	0.28 ^b^	2.17 ^b^	0.17 ^c^	0.25 ^b^	-	-	-	-	-	-	-
*H. opuntiae* GM10	0.16 ^b^	3.33 ^c^	1.85 ^d^	0.21 ^b^	-	-	-	-	0.30 ^b^	-	-
*H. uvarum* GM19	1.60 ^c^	8.07 ^a^	2.51 ^b^	0.58 ^c^	0.23 ^b^	0.49 ^c^	0.23 ^b^	0.50 ^c^	-	-	0.86 ^c^
*H. lanchancei* GM32	-	-	-	-	-	-	-	-	-	-	-
*H. pseudoguiller* GP14	0.30 ^a^	2.50 ^b^	0.14 ^c^	0.38 ^b^	-	-	-	-	-	-	-
*H. guilliermondii* GA1	-	0.35 ^d^		0.59 ^c^	0.25 ^b^	0.26 ^b^	0.18 ^b^	0.45 ^c^	-	-	0.11 ^b^
*C. awuaii* GM3	-	0.15 ^d^	-	0.58 ^c^	0.18 ^b^	-	-	0.12 ^b^	-	-	-

* Under the detection limit (<0.1 ppm eq). The letters (a–c) show mean comparison analysis and same letters means no statistically significant difference between the yeast inhibition zone.
